# Cervical intradural disc herniation: a new diagnostic clue based on MRI T2 hyperintensity and intraoperative increased water content in the intervertebral disc—a case report and mechanistic insights

**DOI:** 10.3389/fsurg.2025.1653174

**Published:** 2025-11-27

**Authors:** Guodong Guo, Xiaohui Shi, Yang Qiu, Daokuan Gao, Peng Fang, Huijuan Ma, Gang Liu

**Affiliations:** 1 Department of Orthopaedics, Nanjing Jinling Hospital, Affiliated Hospital of Medical School, Nanjing University, Nanjing, China; 2 Department of Neurology, Sir Run Run Hospital, Nanjing Medical University, Nanjing, China

**Keywords:** cervical intradural disc herniation, MRI T2 hyperintensity, increased water content, Brown-Séquard syndrome, anterior cervical corpectomy and fusion

## Abstract

**Background:**

Cervical intradural disc herniation (CIDH) is a rare spinal condition characterized by herniation of nucleus pulposus through the dura mater into the subarachnoid space, resulting in spinal cord or nerve root compression. Its rarity and nonspecific imaging features make preoperative diagnosis particularly challenging.

**Case presentation:**

We report a 55-year-old man with sudden-onset left-sided limb weakness and inability to walk, without prior trauma. Neurological examination revealed Brown-Séquard syndrome, with ipsilateral motor deficits and contralateral sensory loss. Sagittal T2-weighted MRI demonstrated marked hyperintensity at the C4/5 intervertebral disc compared to adjacent levels. Intraoperatively, the disc material exhibited visibly increased water content compared to typically desiccated degenerative discs. A small amount of clear fluid was noted near the posterior longitudinal ligament. Further exploration revealed a ventral dural tear with herniated disc fragments within the subarachnoid space, confirmed histologically as CIDH. The patient underwent anterior cervical corpectomy and fusion with dural repair, resulting in neurological recovery. At one-year follow-up, only mild residual weakness in the left hand remained.

**Conclusion:**

This case underscores T2 hyperintensity and intraoperative detection of disc tissue with increased water content as potential indicators of CIDH in acute cervical myelopathy. Recognizing these features may support earlier diagnosis, guide surgical planning, and reduce complications such as cerebrospinal fluid leakage.

## Introduction

Cervical intradural disc herniation (CIDH) is an uncommon spinal disorder characterized by penetration of nucleus pulposus through the dura mater into the subarachnoid space, causing direct compression of the spinal cord or nerve roots. CIDH accounts for less than 0.3% of all disc herniations, with cervical cases comprising only about 3% of intradural occurrences (1). Since its initial description by Marega in 1959, fewer than 50 confirmed cases have been reported worldwide as of 2025 (2–4). Owing to its rarity, nonspecific imaging features, and limited clinical awareness, preoperative diagnosis remains challenging, with an estimated detection rate of only 13% (2).

We report a spontaneous case of CIDH in a middle-aged man presenting with acute Brown-Séquard syndrome. T2-weighted MRI showed marked hyperintensity at the C4/5 disc, and intraoperatively, the disc material demonstrated increased water content compared to typical degenerative discs.These features may represent a previously underrecognized diagnostic clue. In cases of acute and severe cervical myelopathy with similar radiologic and intraoperative findings, surgeons should maintain a high index of suspicion for CIDH and prepare for potential dural tears and cerebrospinal fluid leakage during preoperative and intraoperative planning.

## Case presentation

**Figure 1:**
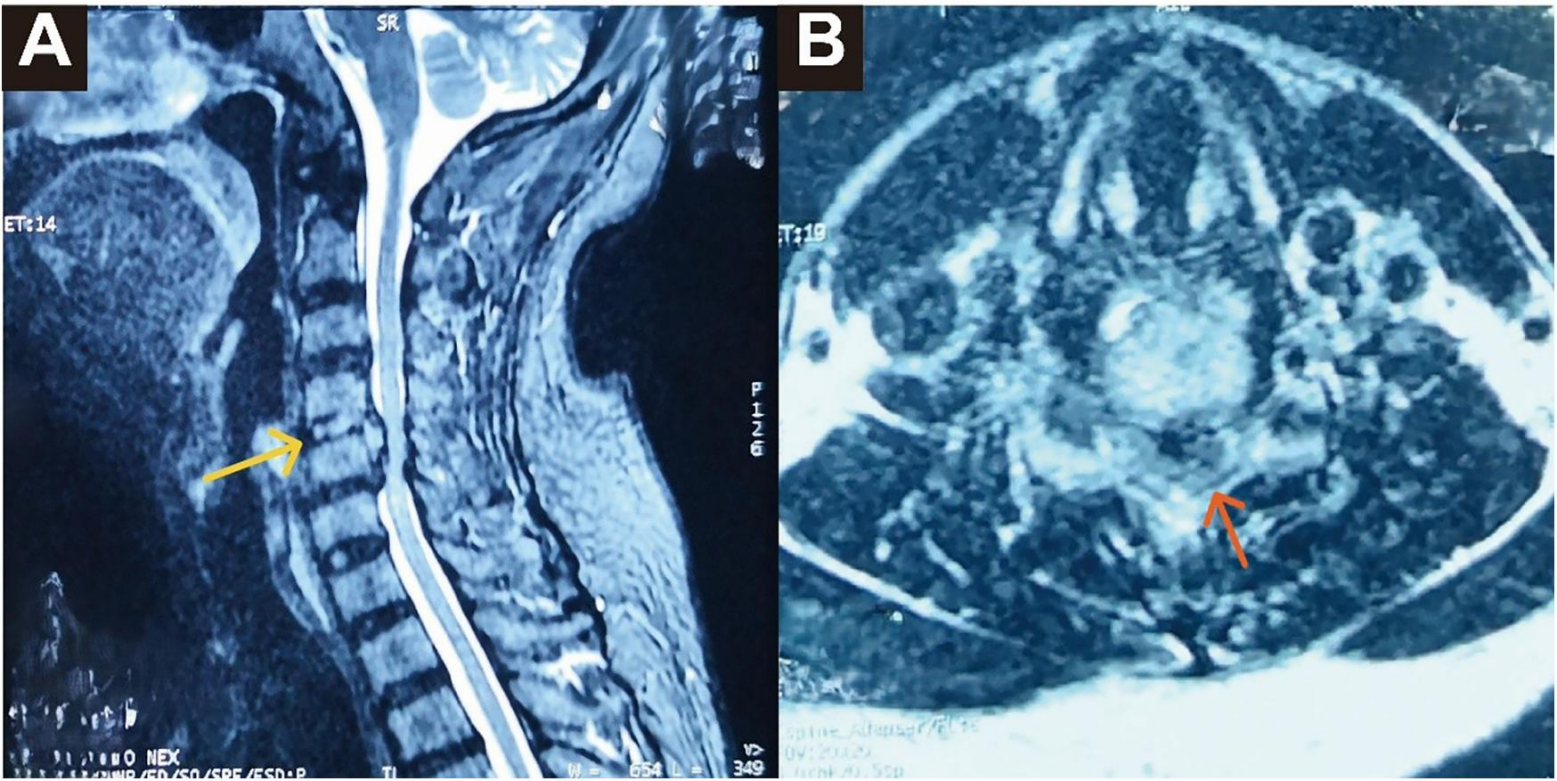
Preoperative MRI. **(A)** Sagittal T2-weighted MRI demonstrates a markedly hyperintense signal at the C4/5 intervertebral disc compared to adjacent discs. A space-occupying lesion is seen posterior to the C5 vertebral body, with both the disc space and the lesion appearing hyperintense. The corresponding segment of the spinal cord is significantly compressed (yellow arrow). **(B)** Axial T2-weighted image demonstrating irregular left posterolateral disc extrusion compressing the cervical spinal cord (orange arrow).

A 55-year-old man presented with sudden-onset limb weakness lasting one day, without any identifiable precipitating event. He had been ambulatory the day prior. After excluding intracranial pathology, he was referred to the orthopedic department. On admission, he demonstrated significant weakness in the left extremities and was unable to ambulate independently. Neurological examination revealed asymmetric motor deficits with ASIA motor scores (right/left): Biceps 4/3, Wrist extensors 4/3, Triceps 4/3, Finger flexors 3/2, Finger abductors 3/2, Iliopsoas 4/0, Quadriceps 4/2, Tibialis anterior 4/2, Extensor hallucis longus 4/2, Gastrocnemius 4/2. Sensory testing showed complete loss of pain and temperature sensation on the right side. Deep tendon reflexes—including triceps, patellar, and Achilles—were absent bilaterally. No clonus or Hoffman's sign was present, and sphincter function remained intact. The presentation was consistent with Brown-Séquard syndrome. Sagittal T2-weighted MRI revealed marked hyperintensity at the C4/5 disc compared to adjacent levels, with a space-occupying lesion posterior to the C5 vertebral body causing significant spinal cord compression (Figure 1). The thoracic and lumbar spine appeared normal. Cervical CT showed no evidence of ossification of the posterior longitudinal ligament (OPLL), and no obvious abnormalities were observed in the vertebral body bone. Preoperative and postoperative hematological and inflammatory markers showed no evidence of infection.

**Figure 2:**
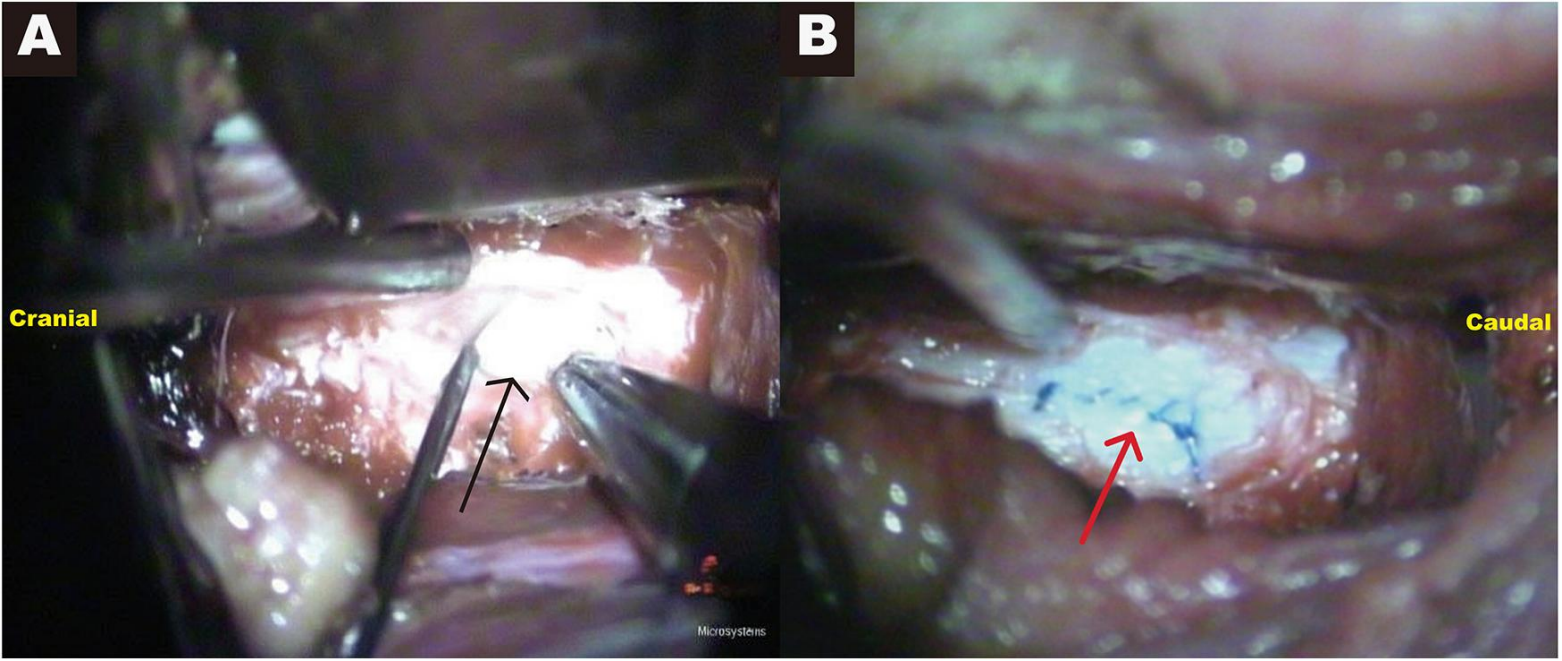
Intraoperative findings. **(A)** Intraoperative view of a longitudinal tear along the ventral midline dura mater, with free disc fragments being extracted through the defect (black arrow). **(B)** Primary dural closure performed using direct suturing (red arrow).

**Figure 3:**
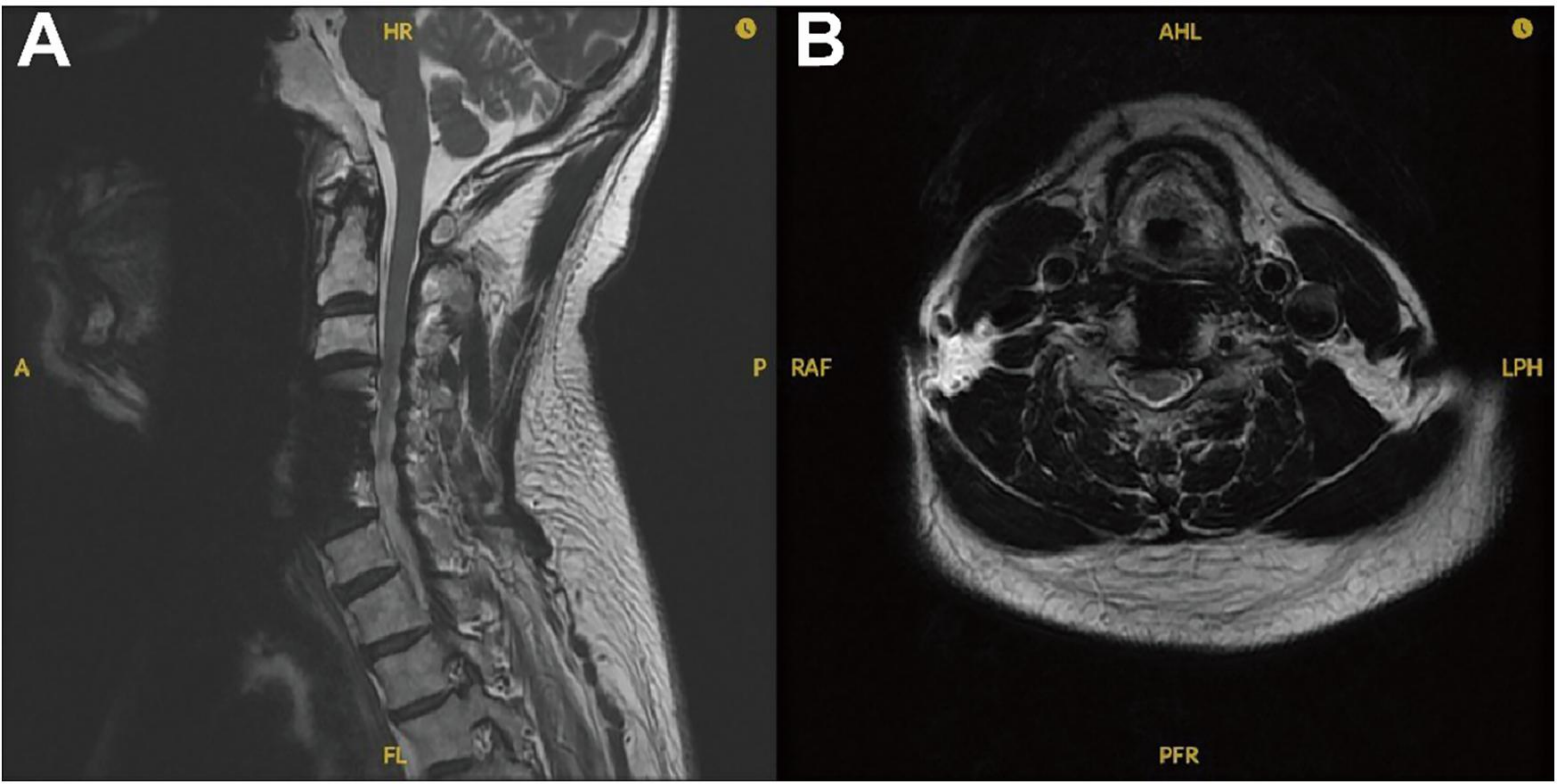
MRI at 3-month follow-up. **(A)** Sagittal and **(B)** axial T2-weighted images show the postoperative status following ACCF. The cerebrospinal fluid line is restored, and spinal cord compression has resolved.

A preliminary diagnosis of cervical disc herniation was made. Due to rapid neurological decline, emergency anterior decompression was performed. Partial resection of the anterior longitudinal ligament and C4/5 disc exposed disc tissue with notably increased water content, contrasting with the typically dry, fibrotic appearance of degenerative discs. Upon reaching the level of the posterior longitudinal ligament, a small amount of clear fluid was observed, but no obvious compressive material was identified, raising suspicion for CIDH. Due to limited visualization, subtotal C5 corpectomy was performed. Intraoperative exploration revealed a longitudinal ventral dural tear, approximately 2 cm in length, through which disc fragments had herniated into the subarachnoid space. The dural margins were defined, the tear gently expanded, and the intradural fragments were removed under microscopic guidance (Figure 2). Histopathological examination confirmed the tissue to be nucleus pulposus, with an oval-like shape and a maximum diameter of approximately 1 cm. Primary dural repair was achieved with sutures (Figure 2), augmented with fibrin glue and autologous subcutaneous cervical fat. The procedure was completed with anterior cervical corpectomy and fusion (ACCF). Postoperative rehabilitation led to substantial recovery. At three months post-operation, following rehabilitation therapy, the patient was able to walk with a cane, indicating initial functional recovery (Figure 3). At one-year follow-up, motor strength had returned to grade 5 except for mild residual weakness in the intrinsic muscles of the left hand.

## Discussion

This case, combined with a literature review, focuses the discussion on three key aspects: clinical and radiological features, the pathophysiological basis of T2 hyperintensity and intraoperative findings, and surgical considerations.

### Clinical presentation and diagnostic challenges

The patient presented with acute-onset Brown-Séquard syndrome, aligning with previous reports where 43.2%–56.5% of CIDH patients exhibit lateralized spinal cord compression, and presenting with quadriplegia is the second most common clinical manifestation, accounting for 34.8% of cases (2, 5). Due to its rarity and nonspecific clinical manifestations, the preoperative diagnostic rate is reportedly as low as 13%, highlighting the need for more reliable diagnostic indicators.

While prior literature describes radiologic signs such as the halo sign, Y sign, and Hawk-beak sign, these findings often lack sensitivity and may be absent in early or atypical presentations (6–8). In our case, such classic signs were not observed. Instead, two notable findings were present: marked T2 hyperintensity at the affected disc level and intraoperative identification of disc tissue with increased water content. These features may serve as novel indirect clues for CIDH, especially in patients presenting with acute cervical myelopathy, no trauma history, and significant cord compression. Early recognition may facilitate accurate preoperative diagnosis and allow for adequate dural exposure, reducing the risk of missed intradural fragments and unintended cerebrospinal fluid (CSF) leakage.

The imaging features of cervical intradural disc herniation (CIDH) can mimic those of various intradural extramedullary lesions, necessitating a comprehensive evaluation of clinical history, imaging characteristics, and laboratory findings for accurate differential diagnosis. The main differential diagnoses include: (1) intradural extramedullary tumors, which typically present with progressive spinal cord compression and demonstrate contrast enhancement on MRI; (2) spinal hematoma or abscess, characterized by acute onset, often with fever or elevated inflammatory markers—hematomas usually exhibit time-dependent signal changes on MRI, whereas abscesses may show peripheral enhancement; and (3) spinal metastases, which can produce intradural lesions and spinal cord compression, usually accompanied by a history of primary malignancy and systemic symptoms (e.g., weight loss, fatigue), and may manifest as multiple lesions on imaging.

### Pathophysiological basis of T2 hyperintensity and intraoperative increased water content

On T2-weighted MRI images, a cerebrospinal fluid signal surrounding the herniated disc—known as the “halo sign”—may be observed (6, 9). This finding suggests that disc material has penetrated the dura mater and entered the subarachnoid space, becoming encased by CSF. First described by Borm (6), the halo sign has since been validated by subsequent studies as a valuable indicator of CIDH (5, 10). Based on this, we hypothesize that dural tearing combined with CSF reflux and infiltration underlies the characteristic findings in our case. The dural defect likely allowed CSF to permeate the herniated disc material or adjacent tissues, increasing local water content and resulting in pronounced T2 hyperintensity. Intraoperative observation of moist disc tissue and the presence of a small amount of clear fluid supports this hypothesis, suggesting that CSF may have mixed with the nucleus pulposus through the dural tear, thereby altering the physical properties of the affected tissue.

### Surgical considerations

In the surgical management of CIDH, addressing the dural defect and ensuring complete decompression are paramount. The incidence of intraoperative cerebrospinal fluid leakage is notably high, ranging from 50% to 70%, underscoring the importance of meticulous dural repair (11). Techniques such as microsurgical suturing, augmented with autologous grafts and fibrin sealant, are essential for achieving a watertight closure, as exemplified in this case (2). Furthermore, when CIDH is suspected or when disc material with increased water content is encountered intraoperatively, a thorough exploration of the intradural space is necessary. This ensures the identification and excision of all intradural disc fragments and allows for the verification of dural integrity, thereby minimizing the risk of postoperative complications such as persistent CSF leakage or recurrent compression.

Spinal stabilization is another critical component of the surgical approach for CIDH, particularly when extensive decompression is required. Subtotal corpectomy may be necessary to adequately expose ventral dural defects, especially in cases where the herniation is located posterior to the vertebral body or when visualization is limited (12). Following decompression and dural repair, anterior fusion is typically performed to maintain spinal alignment and stability. This step is crucial for preventing postoperative instability and promoting long-term recovery, as demonstrated in this case where anterior C5 corpectomy and fusion were successfully employed. By addressing both the neural decompression and spinal stability, the surgical strategy ensures optimal outcomes for patients with this rare but challenging condition.

## Conclusion

Cervical intradural disc herniation remains a rare and diagnostically challenging entity due to its nonspecific clinical and radiological features. This case underscores the diagnostic relevance of disc-level T2 hyperintensity and intraoperative identification of disc tissue with increased water content as potential surrogate markers of dural penetration. Early recognition of these features may enhance preoperative suspicion and guide surgical planning. Intraoperative dural repair and thorough decompression are critical for achieving favorable outcomes, while anterior fusion is essential for maintaining spinal stability following extensive ventral exposure. Increased awareness of these diagnostic and surgical considerations may facilitate earlier detection and optimize the management of this rare but clinically significant condition.

## Data Availability

The original contributions presented in the study are included in the article/Supplementary Material, further inquiries can be directed to the corresponding authors.
